# Immune response of the Caribbean sea fan, *Gorgonia ventalina*, exposed to an *Aplanochytrium* parasite as revealed by transcriptome sequencing

**DOI:** 10.3389/fphys.2013.00180

**Published:** 2013-07-25

**Authors:** Colleen A. Burge, Morgan E. Mouchka, C. Drew Harvell, Steven Roberts

**Affiliations:** ^1^Department of Ecology and Evolutionary Biology, Cornell UniversityIthaca, NY, USA; ^2^School of Aquatic and Fishery Sciences, University of WashingtonSeattle, WA, USA

**Keywords:** *Gorgonia ventalina*, sea fan, transcriptome, RNA-Seq, soft coral, immune response

## Abstract

Coral reef communities are undergoing marked declines due to a variety of stressors including disease. The sea fan coral, *Gorgonia ventalina*, is a tractable study system to investigate mechanisms of immunity to a naturally occurring pathogen. Functional studies in *Gorgonia ventalina* immunity indicate that several key pathways and cellular components are involved in response to natural microbial invaders, although to date the functional and regulatory pathways remain largely un-described. This study used short-read sequencing (Illumina GAIIx) to identify genes involved in the response of *G. ventalina* to a naturally occurring *Aplanochytrium* spp. parasite. *De novo* assembly of the *G. ventalina* transcriptome yielded 90,230 contigs of which 40,142 were annotated. RNA-Seq analysis revealed 210 differentially expressed genes in sea fans exposed to the *Aplanochytrium* parasite. Differentially expressed genes involved in immunity include pattern recognition molecules, anti-microbial peptides, and genes involved in wound repair and reactive oxygen species formation. Gene enrichment analysis indicated eight biological processes were enriched representing 36 genes, largely involved with protein translation and energy production. This is the first report using high-throughput sequencing to characterize the host response of a coral to a natural pathogen. Furthermore, we have generated the first transcriptome for a soft (octocoral or non-scleractinian) coral species. Expression analysis revealed genes important in invertebrate innate immune pathways, as well as those whose role is previously un-described in cnidarians. This resource will be valuable in characterizing *G. ventalina* immune response to infection and co-infection of pathogens in the context of environmental change.

## Introduction

Infectious diseases, caused by a variety of pathogens, are contributing to the decline of coral reefs worldwide (reviewed by Sutherland et al., 2004; Harvell et al., 2007; Bourne et al., 2009) by threatening biodiversity, causing marked population declines, and changing community structure (Harvell et al., 2002). Corals, like other invertebrates, defend against pathogenic invaders using the innate immune system, an ancient defense system found in both invertebrates and vertebrates. In cnidarians, both genomic and functional studies indicate the existence of key innate immune components including pathogen recognition, signaling cascades, and effector responses [reviewed by (Miller et al., 2007; Dunn, 2009; Augustin and Bosch, 2010; Palmer and Traylor-Knowles, 2012)]. Targeted approaches suggest a key role of immune receptors (e.g., modified Toll like receptors, (Bosch et al., 2009) anti-microbial peptides (e.g., Bosch et al., 2009; Vidal-Dupiol et al., 2011), and the inflammatory cascade (Mydlarz et al., 2008) in the cnidarian response to pathogens.

The sea fan coral, *Gorgonia ventalina*, underwent large-scale declines in the Caribbean in the 1990s (reviewed by Burge et al., 2013) as a result of “Aspergillosis” caused by the fungal pathogen *Aspergillus sydowii* (Smith et al., 1996; Geiser et al., 1998). A second sea fan pathogen, an *Aplanochytrium* spp, a marine stramenopile protist (Order Labyrinthulomycetes) was also recently described, isolated, and cultured, (Burge et al., 2012a) where clear damage to the host has been noted in association with Labyrinthulomycete cells including longitudinal tearing of the host gorgonin (skeleton) and degradation of the host polyps (Burge et al., 2012a, 2013). The well-characterized disease ecology and the identification and culture of multiple natural pathogens has made the sea fan one of the best-studied corals the in the context of host immunity (e.g., Kim and Harvell, 2004; Mydlarz et al., 2008). To date, the response to pathogen exposure or disease in *G. ventalina* has focused on measurement of effector enzymes (e.g., prophenoloxidase, peroxidase, chitinase, catalase, and antifungal and antibacterial peptides) (Douglas et al., 2007; Mydlarz and Harvell, 2007; Couch et al., 2008; Mydlarz et al., 2008) and pathological responses using histology (Petes et al., 2003; Mydlarz et al., 2008; Burge et al., 2012a). These studies demonstrate cellular and systemic responses that play a critical role in sea fan immune function. For instance, the inflammatory response of amoebocytes to infections in *G. ventalina* includes production of prophenoloxidase enzymes that enable the formation of a melanin barrier within the sea fan skeleton (Mydlarz et al., 2008), which is the primary observed pathological response of sea fans to both fungal and Labyrinthulomycete infection (Petes et al., 2003; Mydlarz et al., 2008; Burge et al., 2012a). While functionally important and supported by studies in other invertebrates, these effector responses are a limited portion of the sea fan immune response and it is unclear what pathways and regulatory networks are ultimately responsible for their initiation.

A transcriptomics approach employed to study sea fan immune physiology would provide a comprehensive understanding of how sea fans recognize and respond to pathogens, information that will expand our knowledge of innate immunity in cnidarians. The aims of this study were to generate a transcriptome for the sea fan coral and to characterize the sea fan host immune response to the *Aplanochytrium* spp. (24 h post exposure) using RNA-Seq analysis. The transcriptome data generated for *G. ventalina* will enable future studies focusing on key functional aspects of sea fan immunology, including environmental drivers of disease and host immunity, co-infection dynamics mediated by the immune system, and mechanisms of immune priming.

## Methods and materials

### Sea fan collection and husbandry

Twelve *G. ventalina* individuals were collected at Laurel Patch Reef, La Parguera, Puerto Rico (17° 56.608′ N, 67° 03.208′ W) in May 2010. Sea fans were cut into two, 6 × 9 cm pieces, and suspended *in situ* to heal on the reef for 3 days. Sea fans were then collected from the reef and moved into static (non-flow through) 38 L aquariums with water circulation and aeration at the University of Puerto Rico, Isla Magueyes Laboratories in La Parguera, PR. Using a clonally replicated design, duplicate sea fan pieces were distributed equally between six aquaria, with half of the pieces placed in 3 control aquaria, and the other half placed within 3 treatment aquaria, for a total of 4 sea fan fragments per aquarium. The aquaria were placed within a wet-table with natural lighting. Temperature (28–32°C), and salinity (35 ppt) were similar to *in situ* measurements made on the reef and the incoming sea water to the marine laboratory (data not shown) throughout the experiment. Temperature, salinity, and light levels were similar across the wet table (data not shown). Sea fans were acclimated for 2 days and water was changed twice daily.

### Experimental inoculations

One piece of each sea fan (*n* = 12) was injected three times (~4000 cells per injection point or 100 μl), ~3 cm apart, just under the surface of sea fan tissue, into the central axis, with a solution of a 5 day culture of an *Aplanochytrium* previously isolated from a sea fan (Burge et al., 2012a). Because of the opportunistic nature of the Labyrinthulomycete infection, it is thought that damage of tissue and/or the gorgonin skeleton enables pathogen entry (Burge et al., 2013). Thus, injection is likely the best mimic of pathogen entry into the host. Complementary clonal pieces (*n* = 12) were injected with QPX media only (Kleinschuster et al., 1998) to serve as a control. After 24 h the areas surrounding each of the three injection points cut from the sea fan, and flash frozen in liquid nitrogen and subsequently stored at −80°C. Sea fan samples were shipped on dry ice overnight to Cornell University and stored at −80°C until sample preparation.

### Sample preparation

Each individual sea fan sample was ground in liquid nitrogen using a mortar and pestle, and the resulting powder was placed in a 2.0 mL microcentrifuge tube. Total RNA was extracted using a modified Trizol/Qiagen RNeasy protocol, whereby following the ethanol precipitation step of the Trizol manufacturer's instructions (Invitrogen, The Life Technologies Corporation™, Grand Island, NY), the aqueous solution was added to an RNeasy column, and the Qiagen RNeasy manufacturer's instructions were subsequently followed (Qiagen, Valencia, California). DNA was removed from extracted RNA using the Turbo DNA-free treatment according to the manufacturer's instructions (Ambion Inc., The Life Technologies Corporation™, Grand Island, NY). Removal of DNA was confirmed by using RNA (1 μl) as template in a quantitative Polymerase Chain Reaction (qPCR) targeting 18 s ribosomal DNA as previously described (Burge and Friedman, 2012). RNA concentrations were quantified using the NanoDrop® ND-1000 (NanoDrop Technologies, Wilmington, DE).

### cDNA library preparation and sequencing

For each treatment (*Aplanochytrium* exposed and control, respectively), twelve samples were pooled using 900 ng of total RNA from each sea fan. RNA quality was assessed using an Agilent BioAnalyzer 2100 at the Cornell University Life Sciences Core Laboratory Center (CLC). Library preparation was done at the Cornell Microarray facility using the mRNA-Seq 8-Sample Prep Kit (Illumina, San Diego, California) followed by sequencing preparation using the standard cluster generation kit and 36 cycle Illumina sequencing kit (Illumina, San Diego, California) at the Cornell University CLC. Each library was sequenced in its own individual lane where 86 bp reads were captured using an Illumina/Solexa Genome Analyzer at the Cornell University CLC. The data discussed in this publication have been deposited in NCBI's Gene Expression Omnibus (Edgar et al., 2002) and are accessible through GEO Series accession number GSE40169 (http://www.ncbi.nlm.nih.gov/geo/query/acc.cgi?acc=GSE40169).

### Transcriptome assembly

Initially, all sequences were trimmed based on quality scores of 0.05 (Phred; Ewing and Green, 1998; Ewing et al., 1998) and the number of ambiguous nucleotides (>2 on ends). Sequences smaller than 20 bp were also removed. *De novo* assembly was carried out using combined reads from both the *Aplanochytrium* exposed and control libraries using CLC Genomics Workbench v4.0 (CLC Bio) with the following parameters: similarity = 0.90, length fraction = 0.8, insertion cost = 3, deletion cost = 3, mismatch cost = 2 and minimum size = 400.

### Transcriptome completeness

To characterize the completeness of the *G. ventalina* transcriptome assembly and assess similar gene families across related species, the *G. ventalina* transcriptome was compared to other cnidarian transcriptomes. A blast search (<1E-5) was performed comparing the *G. ventalina* transcriptome to *N. vectensis* [27, 273 sequences; *Nematostella vectensis* genome project (2012)] and *A. millepora* [95, 400 sequences; (*Acropora millepora* transcriptome (2012)]. Furthermore, similarly as previously described (Polato et al., 2011) we used OrthoDB (Waterhouse et al., 2011) to identify orthologs conserved in metazoans and single copy in *Hydra magnipapillata* and *N. vectensis* to compare with the *G. ventalina* transcriptome. Specifically we used 118 conserved proteins (across metazoans) from *H. magnipapillata* and *N. vectensis* to identify *G. ventalina* homologs using TBLASTN.

### Transcriptome annotation

Consensus sequences (or contigs) were compared to the UniProtKB/Swiss-Prot database. Comparisons were made using the BLASTx algorithm (Altschul et al., 1990) with a maximum of 1E-6 *e*-value threshold. Swiss-Protein identifiers were joined to the associated GO terms (Gene Ontology database: http://www.geneontology.org) to categorize genes into parents categories and to functional group based on the MGI GO Slim database (http://www.informatics.jax.org) using the table joining feature in Galaxy (Blankenberg et al., 2010; Goecks et al., 2010).

Unable to comprehensively exclude non-host sequence, we also compared our contigs to an available Labyrinthulomycetes resource, the *Aurantiochytrium limacinum* genome sequencing at the NCBI Short Read Archive under accession number PRJNA68529 using an *E*-value threshold of 1E-40.

### RNA-Seq analysis

RNA-Seq analysis was performed to determine differential gene expression patterns between *Aplanochytrium*-exposed and control libraries. RNA-Seq allows for deep sequencing and quantitative analysis of short cDNA reads (Wang et al., 2009). CLC Genomics Workbench v4.0 (CLC Bio) was used to map the reads to the assembled transcriptome and to obtain raw counts of sequencing reads using following parameters: unspecific match limit = 5, maximum number of mismatches = 2, minimum number of reads = 10. Statistical comparisons of count data between the control and *Aplanochytrium*-exposed libraries were carried out using DESeq (Anders and Huber, 2010). DESeq normalizes the count data (based on library size) and calculates mean values, fold change, size factors, and raw and adjusted *p*-values using a generalized linear model with a negative binomial distribution and shrinkage estimator. Only FDR adjusted *p*-values (standard adjusted *p*-values using DESeq) were used in the subsequent analysis. Genes were considered differentially expressed in a given library when the adjusted *p*-value was less than or equal to 0.05. All genes described had respective *e*-values of less than 1E-6 except for selected immune related genes. For these genes, nucleotide sequences were translated using ORF finder (Tutusov and Tutusov, 2011) and compared to the Swiss-Prot database using a p-blast, psi-blast, and pfam-searches (Altschul et al., 1997; Finn et al., 2010) Only sequences with significant protein domain (<E-6) similarity were considered.

To identify enriched biological themes and GO terms in our differentially expressed genes, the Database for Annotation, Visualization and Integrated Discovery (DAVID) v6.7 was used (Huang et al., 2009a,b). The Swiss-Pro accession numbers for the assembled transcriptome were used as a background, and corresponding Swiss-Pro accession numbers for the differentially expressed genes as the gene list. Biological processes (corresponding to specific genes) were considered significantly enriched with a *p*-value < 0.05, and data generated include the identity and number of genes associated with a specific process, and the fold “enriched” of a specific process as compared to the transcriptome.

### Corroboration of illumina sequencing approach by reverse transcriptase quantitative-PCR

RNA extracted from six 6 control and 6 *Aplanochytrium*-exposed sea fan fragments was used for real time quantitative-PCR (RT-qPCR) validation of Illumina sequencing. RNA samples were not pooled for RT-qPCR and were run on replicate individuals. First-strand cDNA synthesis was performed using 1 μg of total RNA, and the GoScript™ Reverse Transcription System (Promega, Madison, WI) according to the manufacturer's protocol. cDNA was diluted 5-fold and used as template in qPCR reactions. qPCR primers were designed using Primer 3 (Rozen and Skaletsky, 2000) for eight genes (Table S1). All qPCR reactions (25 μl), including no template controls run with nuclease-free water, were performed in duplicate in 96-well microplates containing 12.5 μl Fast SYBR® Green Master Mix (Applied Biosystems, The Life Technologies Corporation™, Grand Island, NY), 300 nm of each primer, and 2 μl of cDNA. All RT-qPCR reactions were carried using an ABI Step One Plus Real Time PCR System (Applied Biosystems, The Life Technologies Corporation™, Grand Island, NY), and the following reaction conditions: 95°C for 20 s; followed by 40 cycles of 95°C for 3 s and 60°C for 3 s. Following the amplification of sample templates, a melt curve analysis (95°C for 15 s, 60°C for 1:00 min, 55–95°C incremented by 5°C 15 s-1steps) was used to confirm amplification of a single product.

Real-time PCR Miner (Zhao and Fernald, 2005) was used to calculate primer efficiencies and cycle threshold values (Ct) from raw amplification data. Relative expression values for each gene were calculated using the equation 1/(1 + Efficiency)^∧^C_T_. The R0 for each gene was normalized to two controls (elongation factor 1 and Polyadenylate-binding protein), except for elongation factor 1 and polyadenylate-binding protein, which were normalized to polyadenylate-binding protein for the former and elongation factor 1 for the later. Control genes were chosen based on previous work with cnidarians and other invertebrates for which these genes have been shown to be relatively stable. In addition, the relative expression of these genes and their stability was confirmed via RNA-Seq data. Fold change in expression relative to control values was then calculated as the quotients of the above equation. Pearson's Moment Correlation was used to test for a linear relationship between the expression values) from RNASeq and qPCR using R (R Core Team, 2012).

## Results

### Transcriptome assembly

After quality trimming, 36.8 and 34.3 million reads (average length = 84 bp) remained from the *Aplanochytrium*-exposed and control libraries, respectively. *De novo* assembly of reads from both libraries resulted in 90,230 contigs (accessible on FigShare (Burge et al., 2012b) with an average length of 964 bp and an N50 value of 1,149. The average number of reads per contig was 459.6 with an average coverage of 36.8 reads.

### Transcriptome completeness

Blast searches of *G. ventalina* contigs to transcript sets of *N. vectensis* and *A. millepora* indicate 10.7 and 12.1 % of *G. ventalina* contigs had significant matches, respectively. Similarly, a BLAST search comparing *A. millepora* to *N. vectensis* data sets found 13.5% of *A. millepora* contigs with hits. Sequences corresponding to all 118 orthologs conserved across metazoans as identified in OrthoDB (Waterhouse et al., 2011) were identified in the *G. ventalina* transcriptome. Each ortholog matched a unique *G. ventalina* contig with 97.5% of the BLAST comparisons with *N. vectensis* and 94.1% of the comparisons with *Hydra magnipapillata*, having an *e*-value < 1.0E-20.

### Transcriptome annotation

**Figure 1:**
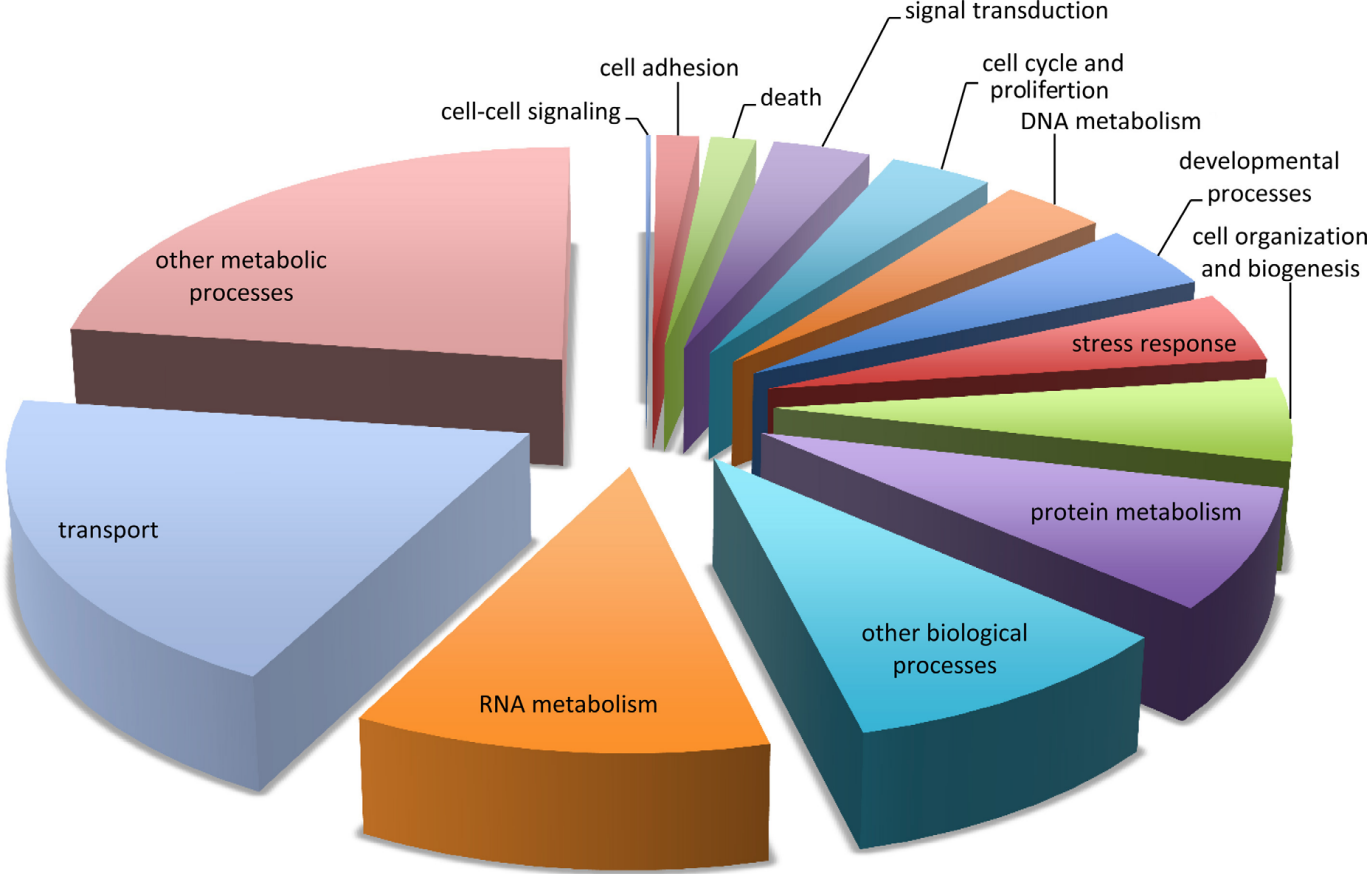
**Functional categorization of the sea fan transcriptome.** Functional categorization of contig sequences from de novo assembly of both libraries using Gene Ontology (GO) biological process “GO slim” classification.

All contigs were compared to the UniProtKB/Swiss-Prot database and 40,142 (44%) of the contig sequences were annotated [accessible on FigShare (Burge et al., 2012b)]. Figure 1 shows associated Gene Ontology (GO) biological process slim terms for the 31,857 contigs that were classifiable. Of particular interest in this study was the identification of genes involved in the stress or immune response. Using GO classification, 1464 contigs were considered associated with the stress response (Figure S1). Immune genes identified by the “stress response” GO Term include components of the complement system (e.g., Complement C2 (Q863A0) and C3 (Q00685 and Q01833), pathogen recognition receptors (e.g. Toll-like receptor 2 (Q9DGB6), 4 (Q9Gl65), and 6 (Q9Y2C9), and scavenger receptor proteins (e.g., Scavenger receptor cysteine-rich type 1 (Q2VL90, P86VB7, and P85521).

A comparison of our transcriptome resource to *Aurantiochytrium limacinum* indicated only 21 sequences with homology based on an *e* < −40. Supplementary File S1 indicates sequences in our transcriptome that may be *Aplanochytrium* in origin.

### RNA-Seq

**Figure 2:**
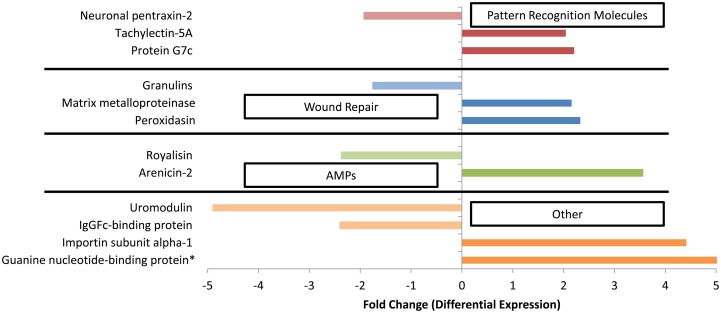
**Differentially expressed genes between *Aplanochytrium*-exposed and control sea fans with possible immune functions (*p* < 0.05).** Positive and negative values represent genes that were up- or down-regulated, respectively, in *Aplanochytrium*-exposed sea fans relative to control sea fans. Expression analysis was performed using DESeq (Anders and Huber, 2010) with annotations based on homology to the Swiss-Protein Database (See Supplementary File 1; http://uniprot.org). ^*^Indicates genes only expressed in sea fans exposed to *Aplanochytrium*.

To determine the transcriptomic response of *G. ventalina* to *Aplanochytrium* spp., RNA-seq analysis was performed. In total, 210 differentially expressed contigs were identified. Of those, 103 were expressed at a higher level in the *Aplanochytrium* exposed sea fans, whereas 107 were expressed at a lower level. Thirty-two percent (or 68) of the differentially expressed contigs (from here on out referred to as genes) were annotated (Supplementary File S2), several of which had putative immune functions such as those involved in pathogen recognition (e.g., Tachylectin-5A, Protein G7c, and Neuronal pentraxin-2), wound repair (e.g., Matrix metalloproteinase or Peroxidasin), antimicrobial peptides (e.g., arenicin-2 and royalisin) (Figure 2, Table S2).

**Figure 3:**
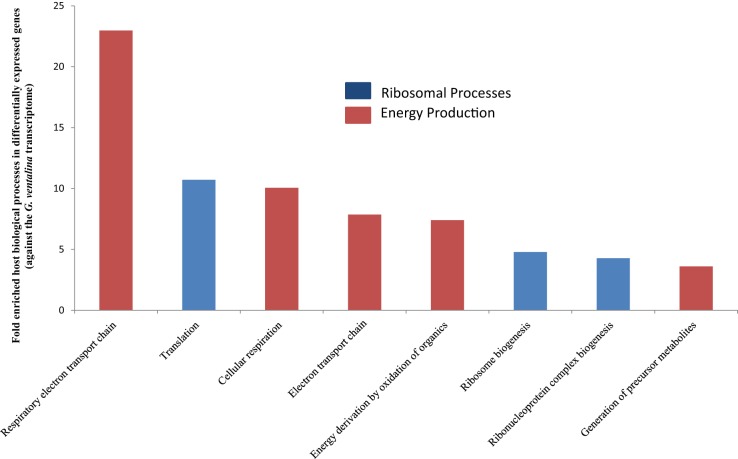
**Gene Ontology (GO) terms for biological processes that were significantly overrepresented in differentially expressed *G. ventalina* genes upon *Aplanochytrium* exposure as determined via enrichment analysis using DAVID (Huang et al., 2009a,b) (*p* < 0.05)**.

Among the 210 differentially expressed genes, 36 genes representing eight GO biological processes were calculated to be significantly enriched based on functional enrichment analysis (as compared to the transcriptome) (*p* < 0.05) (Figure S2; Supplementary File S3). Thirty-five of these 36 genes were up-regulated in the *Aplanochytrium*-exposed sea fans. The biological processes that were enriched are associated with translation of proteins (representing 29 genes, primarily 40S and 60S ribosomal proteins) and energy production in mitochondria (representing 7 genes, e.g., cytochrome c oxidase subunits 1–3, NADH-ubiquinone oxidoreductase chain 1, and ATP synthetase subunit. Categories with the highest fold enrichment include respiratory electron transport chain (~23X), translation (11X), and cellular respiration (10X) (Figure 3).

### Corroboration of illumina sequencing approach by RT qPCR

Expression values determined by the RT-qPCR analysis of eight genes were found to be comparable to the RNA-seq approach (Table S1). We found the values obtained by RT qPCR and RNA-seq were highly similar and correlated with statistical significance (Pearson's Moment Correlation *R* = 0.861, *p*-value = 0.006052) (Figure S3).

## Discussion

Here we report the first transcriptome generated for a gorgonian coral species, *G. ventalina*. We characterized 40,142 contigs based on functional annotation. This data will provide an important resource for further targeted studies on immune physiology of *G. ventalina*. Analysis of the *G. ventalina* transcriptome as compared to other cnidarian transcriptomes (*A. millepora* and *N. vectensis*) indicated a similar number of shared contigs between each cnidarian transcriptome. Additionally, *G. ventalina* contigs included a high percentage of significant matches to orthologs shared across metazoans indicating the transcriptome is relatively complete.

We conducted the first comprehensive transcriptomic characterization of a cnidarian exposed to a natural pathogen. Validation of differentially expressed genes performed using RT-qPCR (on a subset of genes) indicates strong similarity of individual sea fan gene expression using qPCR to our pooled Illumina sequencing data, similar to validation of pooled Illumina results by Altincicek et al., 2013 and qPCR corroboration of other RNA seq studies (e.g., Meyer et al., 2011). We will describe host response based on statistically relevant measures: functionally enriched categories and individual immune genes detected by DESeq analysis.

### Functional enrichment analysis

Based on functional enrichment analysis, the majority of the enriched genes encode ribosomal proteins involved in translation. All of these genes were up-regulated in the *Aplanochytrium*- exposed sea fans, indicating an up-regulation of the host protein response. This pattern has also been documented in other invertebrates' exposed to pathogens [i.e., clams (Gestal et al., 2007); abalone (Travers et al., 2010); urchins (Nair et al., 2006)]. Although the up-regulation of ribosomal gene products is not a specific response to pathogens and is likely a general response to stress, ribosomal proteins may be important regulators of metabolism during pathogen exposure (Travers et al., 2010).

Four of the five categories with the highest fold enrichment include genes primarily involved in production of energy in the mitochondria, indicating an increased energy demand to mount an effective immune response (Nayak et al., 2011) or general stress response. Herein, genes involved in the production of reactive oxygen species (ROS) (NADH-ubiquinone oxidoreductase chain 1 and cytochrome c oxidase subunits) were among enriched processes in sea fans exposed to the *Aplanochytrium*. Additionally, NADH-ubiquinone oxidoreductase chain 1 expression was validated with RT qPCR. Up-regulation of NADH-ubiquinone oxidoreductase 1 and cytochrome c oxidase subunits suggest energy production in the mitochondria through oxidative phosphorylation. In other invertebrate host-pathogen systems, cytochrome oxidases have been shown to be up-regulated in response to immune stimulation [i.e. shrimp: (James et al., 2010); abalone: (van Rensburg and Coyne, 2009); clams: (Gestal et al., 2007)], and in fact, evidence suggests a successful immune response in abalone may depend on electron transport (van Rensburg and Coyne, 2009). Herein, the electron transport chain had the highest fold enrichment (23X). Oxidative phosphorylation also leads to the production and release of ROS, which is an important pathogen-killing mechanism in invertebrate immunity and has been shown to be a critical component of the immune response of many invertebrates, including mosquitoes (Molina-Cruz et al., 2008) and abalone (van Rensburg and Coyne, 2009).

### Immune genes and role in cnidarian immunity

We also identified a number of immune-related genes that were differentially expressed between control and *Aplanochytrium*-exposed sea fans, including those that code for proteins involved in pattern recognition molecules, antimicrobial peptides, and wound repair (refer back to Figure 3). Although these genes were not classified into significantly enriched biological processes, they nonetheless represent genes that play an important role in immune-related processes and should be investigated in more detail. We therefore devote the remaining discussion to these genes and their potential role in cnidarian immunity.

#### Pattern recognition molecules

Pattern Recognition Molecules (PRM), also known as Pattern Recognition Receptors (PRRs), recognize and bind to conserved components of microbial cell walls (e.g., lipopolysaccharide of Gram-negative bacteria). This binding results in a signaling cascade that leads to the induction of effector molecules and cellular components of immunity (Ferrandon et al., 2007; Dunn, 2009). Many of the immune-related genes that were up-regulated (e.g., Tachylectin-5A and Protein G7c) or down-regulated (e.g., Neuronal pentraxin-2) serve as PRMs. Expression of Tachylectin-5A, Protein G7c and Neuronal pentraxin-2 were validated using RT qPCR.

Tachylectin-5a belongs to the fibrinogen related domain (FReD) superfamily. Members of the FReD superfamily have been described in many invertebrate taxa including cephalochordates, and are important in defense processes such as agglutination, pathogen recognition, bacterial lysis, histocompatibility, and parasite defense (reviewed by Hanington and Zhang, 2011). Tachylectin-5A is a plasma lectin isolated from the horseshoe crab, *Tachypleus tridentatus*, and agglutinates human erythrocytes as well as Gram-positive and Gram-negative bacteria (Gokudan et al., 1996). FReD superfamily members are well-studied in mosquito-parasite interactions where RNAi silencing experiments indicate that they are key in clearing bacterial infections (Dong and Dimopoulos, 2009). Furthermore, in snail-trematode interactions, the diversity of FReD superfamily members within individuals has implied somatic diversification (Zhang et al., 2004) and may be associated with resistance (Stout et al., 2009). Tachylectin homologs have been described from EST libraries of anemones and corals (tachylectin-2; Hayes et al., 2010) and *Hydractina* (tachylectin-1), although neither of these putative tachylectin's contain the FReD domain found in Tachylectin 5A or the putative sea fan Tachylectin. While the *Hydractina* tachylectin-like gene has been suggested to play a role in developmental metamorphosis and not immunity (Mali et al., 2006), work by Hayes and colleagues indicates positive selection of *Oculina* tachylectin-2, which is disproportionately observed in immune genes, suggesting an immune-related role for this gene (Hayes et al., 2010). Given that the putative sea fan Tachylectin was up-regulated upon pathogen exposure and possesses a FReD domain, we hypothesize that the sea fan tachylectin functions in host defense.

#### Antimicrobial peptides

Activation of specific PRMs such as the toll like receptors (TLRs) leads to production of antimicrobial peptides in innate immunity. Antimicrobial peptides described in microbial defense in cnidarians include hydramacin-1, arminin 1a, and periculin-1 in Hydra (reviewed by, Augustin and Bosch, 2010), damicornin in a hard coral (Vidal-Dupiol et al., 2011), and aurelin in jellyfish (Ovchinnikova et al., 2006). Transcripts identified in this study are homologous to anti-microbial peptides including arenicin-2 from a polycheate worm which has anti-microbial activity again gram positive, negative, and fungi (up-regulated) (Ovchinnikova et al., 2004), and royalisin from a honeybee which processes activity against gram positive bacteria and fungi (and not against gram negative activity to date) (down-regulated) (Fujiwara et al., 1990). We can speculate that the arenicin-2 homolog is a broader spectrum anti-microbial acting in response to the *Aplanochytrium* cells, while the royalisin homolog may be more involved with normal anti-microbial processes as its expression is higher in the un-exposed sea fans.

#### Wound repair

Cnidarians have a high capacity for regeneration and wound repair, including re-modeling of tissues after physical damage or infection by pathogens [*Hydra*: (Bosch, 2007), (Augustin and Bosch, 2010); *Porites*: (Palmer et al., 2011)]. In Hydra, macromolecules (laminins, collagens, heparin sulfate proteoglycans and fibronectin-like molecules) of the mesoglea (extracellular matrix) are key for regeneration, and wound repair is regulated by metalloproteinases (Bosch, 2007). Matrix metalloproteinases are a major group of enzymes that regulate the cell matrix composition, and ample evidence exists for the role of matrix metalloproteinases (Matrix metalloproteinase-14) in normal and pathological processes (Massova et al., 1998). In response to *Aplanochytrium*, a metalloproteinase was up-regulated. Additionally, the peroxidasin gene, which codes for a protein that acts both in extracellular matrix organization (associated with phagocytosis and defense) and hydrogen catabolic processes (i.e. catalyzing hydrogen peroxide) [*Drosophila*; (Nelson et al., 1994)] [*Xenopus tropicalis*; (Tindall et al., 2005)], was up-regulated.

## Conclusions

Here we report the generation of the first transcriptome for a soft coral species. In addition, we also report the results of the first RNA-seq experiment of a coral exposed to a microbial pathogen. We annotated a large number of immune-related genes, with many of these genes being differentially expressed in response to *Aplanochytrium*, including those involved in pattern recognition, anti-microbial peptide production, and wound repair. Enrichment analysis also identified processes (protein translation and energy production) involved in the host response to *Aplanochytrium* species. With this information, we now have a basis for comprehensively studying the immune response of *G. ventalina* to pathogens.

### Conflict of interest statement

The authors declare that the research was conducted in the absence of any commercial or financial relationships that could be construed as a potential conflict of interest.

## Supplementary material

Supplementary File S1 **Contigs (21) with significant homology (<1E-40) to *Aurantiochytrium limacinum* (SRA: PRJNA68529)**.

Supplementary File S2 **Differentially expressed genes in sea fans exposed to *Aplanochytrium* and the controls**.

Supplementary File S3 **Genes representing the nine functionally enriched GO Biological processes (as compared to the transcriptome)**.

Table S1 **Primer sets used in RT qPCR validation**.

Table S2 **Differentially immune-related genes in seafans exposed to *Aplanochytrium* and their controls**.

Figure S1 **Stress response genes in the sea fan transcriptome. (A)** Go Slim Stress response classes categorized to Go term categories including categories with greater than 10 hits (categories with less than 10 hits summarized as “Other”). GO Terms “Response to DNA Damage (708) “DNA synthesis during DNA Repair (691),” and “Response to Stress (409) are not shown. **(B)** Important innate effectors of the “innate response” and **(C)** Important innate effectors in “inflammation.”

Figure S2 **Enriched host genes: total genes per GO Category.** Analysis of Biological Processes using DAVID (Huang et al., 2009b, a); (*p* < 0.05).

Figure S3 **Correlation of gene expression levels of selected genes by qPCR and RNASeq (Pearson's moment correlation *R* = 0.861, *p*-value = 0.006052)**.
