# Supplementary figures and images for: Immune response of the Caribbean sea fan, Gorgonia ventalina, exposed to an Aplanochytrium parasite as revealed by transcriptome sequencing
Source: Front Physiol. 2013 Jul 25;4:180. doi: 10.3389/fphys.2013.00180 (PMC3722494; doi:10.3389/fphys.2013.00180)

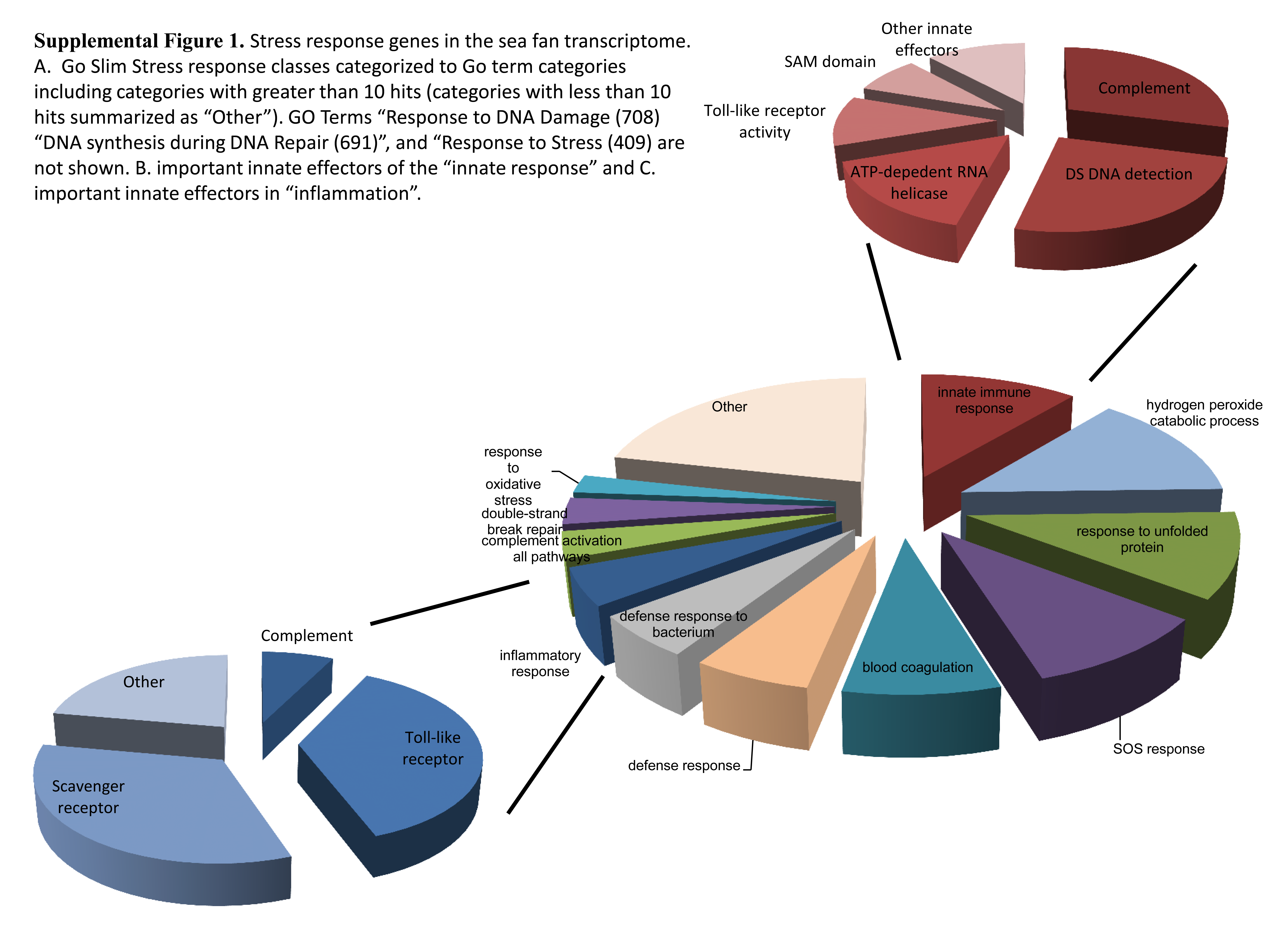

Supplement: Figure S1 — Stress response genes in the sea fan transcriptome. (A) Go Slim Stress response classes categorized to Go term categories including categories with greater than 10 hits (categories with less than 10 hits summarized as “Other”). GO Terms “Response to DNA Damage (708) “DNA synthesis during DNA Repair (691),” and “Response to Stress (409) are not shown. (B) Important innate effectors of the “innate response” and (C) Important innate effectors in “inflammation.” [file Presentation1.ZIP › Supplementary figures/Supp Fig1.tif]

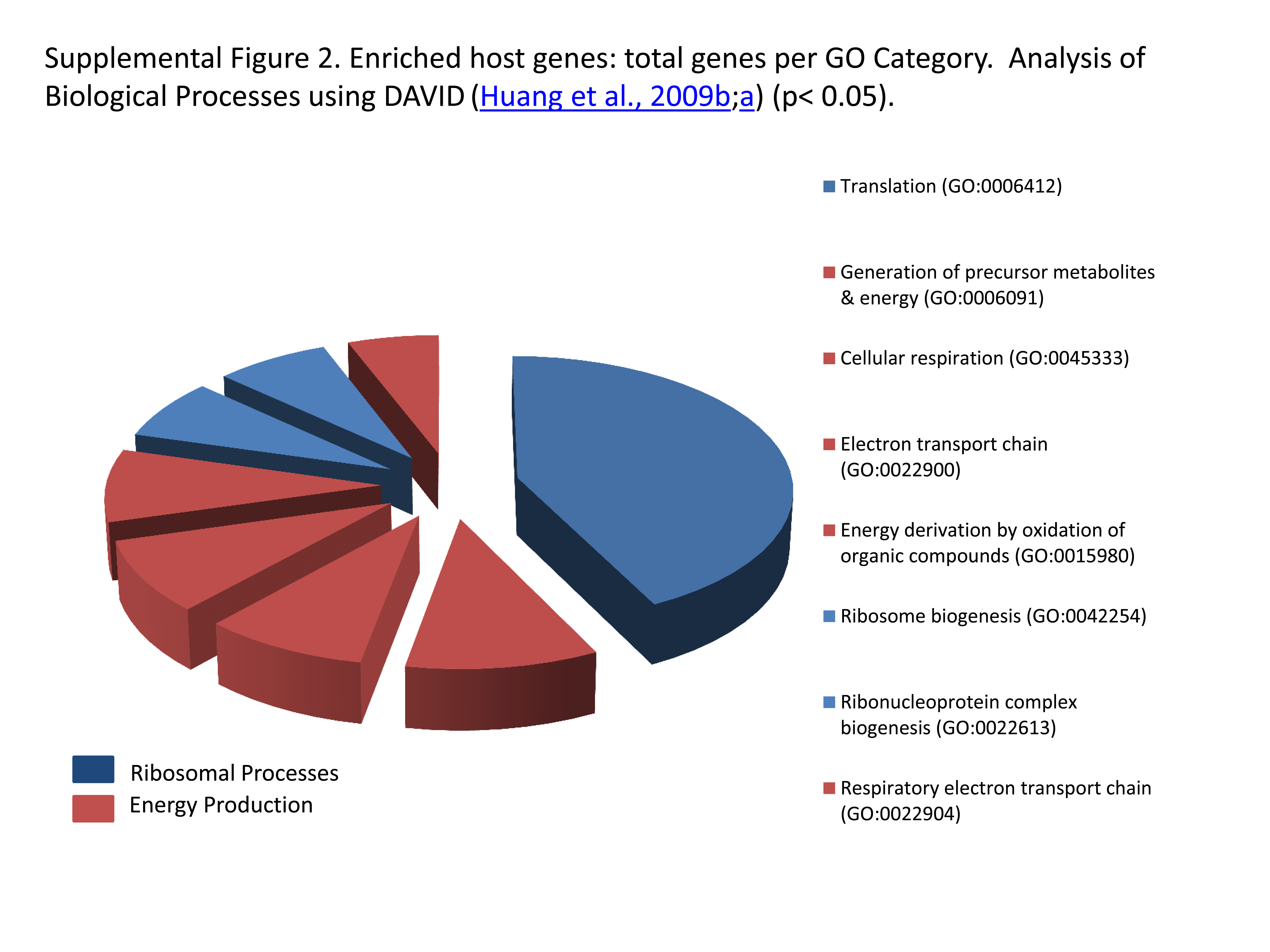

Supplement: Figure S1 — Stress response genes in the sea fan transcriptome. (A) Go Slim Stress response classes categorized to Go term categories including categories with greater than 10 hits (categories with less than 10 hits summarized as “Other”). GO Terms “Response to DNA Damage (708) “DNA synthesis during DNA Repair (691),” and “Response to Stress (409) are not shown. (B) Important innate effectors of the “innate response” and (C) Important innate effectors in “inflammation.” [file Presentation1.ZIP › Supplementary figures/Supp Fig2.tif]

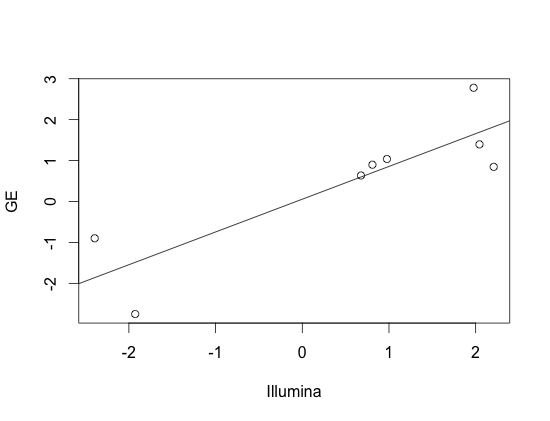

Supplement: Figure S1 — Stress response genes in the sea fan transcriptome. (A) Go Slim Stress response classes categorized to Go term categories including categories with greater than 10 hits (categories with less than 10 hits summarized as “Other”). GO Terms “Response to DNA Damage (708) “DNA synthesis during DNA Repair (691),” and “Response to Stress (409) are not shown. (B) Important innate effectors of the “innate response” and (C) Important innate effectors in “inflammation.” [file Presentation1.ZIP › Supplementary figures/Supp Fig3.tiff]
